# Research progress on the mechanistic pathways and biomarkers of therapeutic drugs for metabolic-associated steatotic liver disease

**DOI:** 10.3389/fcell.2026.1808135

**Published:** 2026-04-23

**Authors:** Lifen Xue, Xin Wang, Jingxin Mao

**Affiliations:** 1 Department of Pharmacy, The First Affiliated Hospital of Chongqing Medical University, Chongqing, China; 2 Technology Development Center, Chongqing Medical and Pharmaceutical College, Chongqing, China; 3 Department of Science and Education, The First Affiliated Hospital of Chongqing Medical and Pharmaceutical College, Chongqing, China

**Keywords:** biomarkers, mechanistic pathways, metabolic-associated steatotic liver disease, metabolic-associatedsteatohepatitis, therapeutic drugs

## Abstract

**Objective:**

The incidence of metabolic-associated steatotic liver disease (MASLD) remains persistently high, and there is a lack of specific therapeutic drugs in clinical practice. Elucidating the mechanistic pathways of therapeutic drugs for MASLD and exploring biomarkers with high specificity and sensitivity are of great significance for the precise diagnosis and treatment of the disease. The present study aims to review the research progress in this relevant field.

**Methods:**

We systematically collated recent domestic and international literature on the mechanistic pathways of therapeutic drugs and biomarkers for MASLD, and analyzed the mechanisms of action, key signaling pathways of different therapeutic drugs, as well as the characteristics of various biomarkers.

**Results:**

Drugs for MASLD treatment mainly exert their effects by regulating lipid metabolism, ameliorating insulin resistance (IR), and inhibiting inflammation and oxidative stress. For instance, peroxisome proliferator-activated receptors (PPARs) agonists improve lipid metabolism and IR via the PPARs signaling pathway; adenosine monophosphate-activated protein kinase (AMPK) activators enhance insulin sensitivity through the AMPK signaling pathway; and anti-inflammatory drugs alleviate inflammatory injury by inhibiting the nuclear factor kappa-B (NF-κB) signaling pathway. Relevant biomarkers include metabolomic biomarkers (lipidome, amino acidome, etc.), inflammatory biomarkers (tumor necrosis factor-α[TNF-α], interleukin-6 [IL-6], etc.), liver fibrosis biomarkers (procollagen type III [Pro-C3], enhanced liver fibrosis [ELF] score, etc.) and non-coding RNA biomarkers, each exhibiting distinct values in disease diagnosis and therapeutic efficacy evaluation.

**Conclusion:**

At present, the core mechanistic pathways of a variety of MASLD therapeutic drugs and potential biomarkers have been identified, yet problems such as insufficient targeting in drug research and development and difficulties in the clinical translation of biomarkers still exist. In the future, it is necessary to further deepen mechanistic research, promote the development of precision therapeutic drugs and the clinical application of biomarkers, so as to provide more reliable theoretical and practical support for the diagnosis and treatment of MASLD.

## 1 Introduction

With the increasing incidence of metabolic diseases such as obesity and type 2 diabetes mellitus (T2DM) worldwide, metabolic-associated steatotic liver disease (MASLD) has become the most common chronic liver disease globally. Its prevalence rate in the general population is as high as more than 25% (Chan et al., 2023), and it is showing an increasing trend year by year, seriously threatening human public health. MASLD is not merely a simple accumulation of fat in the liver, but a complex disease spectrum closely related to various pathophysiological processes such as insulin resistance (IR), lipid metabolism disorders, inflammatory responses, oxidative stress, and intestinal flora imbalance. The disease progression can gradually develop from simple fatty liver to non-alcoholic steatohepatitis, liver fibrosis, and even cirrhosis and hepatocellular carcinoma (HCC), posing great challenges to clinical diagnosis and treatment (Yang et al., 2025). At present, clinical treatment of MASLD is still based on lifestyle interventions, but patients have poor compliance, and there is a lack of unified and effective specific therapeutic drugs. Meanwhile, due to the insidious early symptoms of MASLD, traditional imaging and serological indicators have limited sensitivity and specificity in early disease diagnosis, disease staging, and efficacy evaluation, which are difficult to meet the needs of clinical precise diagnosis and treatment. Therefore, in-depth clarification of the pathogenesis of MASLD, identification of the key mechanistic pathways of therapeutic drugs, and exploration of biomarkers with high specificity and sensitivity (Iwaszko-Sochal et al., 2025) have become research hotspots in the field of hepatology. This article systematically reviews the research progress on the mechanistic pathways of therapeutic drugs and related biomarkers for MASLD, aiming to provide theoretical references and practical strategies for the mechanistic research, drug development, and clinical precise diagnosis and treatment of the disease.

## 2 Overview of metabolic-associated steatotic liver disease

### 2.1 The definition of metabolic-associated steatotic liver disease

MASLD has been renamed from “non-alcoholic fatty liver disease” in recent years, which has been adopted by international multidisciplinary consensus and the latest guidelines in China. The new name emphasizes “metabolic dysfunction” as the fundamental driving factor: the diagnosis can be confirmed when the patient has any one of overweight/obesity, T2DM, or systemic metabolic abnormalities, combined with imaging, serum biomarkers, or liver histology evidence confirming intrahepatic lipid deposition, and excluding other clear causes of liver injury such as alcohol, virus, drugs, and immunity. The disease spectrum presents a continuous progression from simple hepatic fat accumulation to metabolic-associated steatohepatitis (MASH), then to progressive fibrosis, cirrhosis, and even HCC (Rinella and Sookoian, 2024). Its core pathological change is the excessive deposition of triglycerides (TG) in hepatocytes, which subsequently triggers oxidative stress, mitochondrial dysfunction, and innate immune activation, forming a vicious circle of chronic inflammation and impaired insulin signaling pathways. Under long-term action, hepatic stellate cells are activated, collagen is deposited, and eventually, it can evolve into decompensated liver disease, becoming an important cause of end-stage liver failure and liver cancer. Globally, the prevalence of MASLD has exceeded 25% and continues to rise with the epidemic trend of obesity and diabetes; in China, the ultrasound detection rate in the general adult population is about 30%, which has become the first leading chronic liver disease (Canivet et al., 2024). Due to the lack of symptoms in the early stage, most patients have varying degrees of fibrosis at the initial diagnosis, indicating that early screening and intervention are imperative.

### 2.2 Core pathogenesis

The pathogenesis of MASLD is complex and characterized by the interaction of multiple factors. Hepatic lipid metabolism disorder acts as the initiating factor at the core, superimposed with immune microenvironment imbalance and metabolic stress cascade reactions, accompanied by multi-dimensional pathological changes such as abnormal epigenetic modification and organelle dysfunction. These factors jointly drive the progression of the disease from simple steatosis to MASH and liver fibrosis (Feng et al., 2024). Under physiological conditions, the liver maintains lipid homeostasis through fatty acid uptake, *de novo* lipogenesis (DNL), β-oxidation, and very-low-density lipoprotein (VLDL) transport. In the setting of MASLD, this homeostasis is completely disrupted, manifested as the following specific abnormalities: excessive activation of lipid synthesis: the key regulatory pathways of the DNL pathway, including sterol regulatory element-binding protein 1c (SREBP1c) and carbohydrate response element-binding protein (ChREBP), are abnormally activated. For instance, SREBP1c is regulated by the mechanistic target of rapamycin complex 1 (mTORC1) signaling, and the deletion of the Folliculin (FLCN) gene in hepatocytes inhibits its hydrolysis through the TFE3/B-mediated Insig2 pathway, leading to uncontrolled DNL. ChREBP is induced by postprandial high-sugar and high-fat diets, and its activity is enhanced via Kctd17 regulation to promote the conversion of glucose to fatty acids, exacerbating lipid accumulation (Li et al., 2023); impaired fatty acid oxidation: the expression of PPARα, the core regulatory pathway of mitochondrial β-oxidation, is downregulated. In western diet-induced models, the knockout of adenosine triphosphate (ATP) citrate lyase (ACLY) and ACSS genes reduces PPARα levels and inhibits the activity of key oxidative enzymes such as carnitine palmitoyltransferase 1A (CPT1A), thereby decreasing fatty acid catabolism, while intermittent fasting can restore oxidative function by increasing the levels of PPARα ligands; defective lipid transport pathway: the synergistic effect of ApoB, Microsomal Triglyceride Transfer Protein (MTP), and other proteins dependent on VLDL assembly and secretion is impaired. The knockout of the SLRP1 gene, loss-of-function of TM6SF2, and deletion of VMP1 all result in reduced VLDL secretion or synthesis inhibition, triggering hepatic triglyceride accumulation (Ha et al., 2025). On this basis, the polarization imbalance of intrahepatic immune cells and the cascade reaction of metabolic stress become the key drivers for disease progression to MASH. Intrahepatic macrophages (Kupffer cells) polarize toward the pro-inflammatory M1 phenotype and secrete tumor necrosis factor-α (TNF-α), interleukin-6 (IL-6) and other cytokines that exacerbate liver injury, and the high expression of glycoprotein non-metastatic melanoma protein B (GPNMB) in the M2 phenotype is positively correlated with the severity of MASH. Meanwhile, the adipokine network is imbalanced: insufficient secretion of adiponectin (APN) prevents it from activating the adenosine monophosphate-activated protein kinase (AMPK)/peroxisome proliferator-activated receptors (PPARs) pathway through AdipoR1/AdipoR2 receptors to regulate lipid metabolism and exert anti-inflammatory effects, thus accelerating disease progression (He et al., 2025). In addition, disordered epigenetic regulation and organelle dysfunction further participate in the pathological process: abnormal DNA methylation affects the transcription of lipid metabolism-related genes; mitochondrial-endoplasmic reticulum interaction disorders (e.g., Mfn2 downregulation, Mic19 gene knockout) lead to calcium transport imbalance, endoplasmic reticulum stress and mitochondrial dysfunction, which inhibit fatty acid β-oxidation and induce hepatic steatosis.

## 3 Therapeutic drugs for metabolic-associated liver disease

Pharmacotherapy for MASLD centers on regulating metabolism, anti-inflammation and anti-fibrosis, and encompasses multiple drug classes including hypoglycemic agents, PPAR agonists and hormone receptor agonists.

### 3.1 Thiazolidinediones

#### 3.1.1 Core drug: pioglitazone

As a classic thiazolidinedione insulin sensitizer, pioglitazone modulates the expression of genes associated with lipid and glucose metabolism via the specific activation of proliferator-activated receptor γ (PPARγ), serving as a key therapeutic option for MASLD patients with T2DM. Its clinical efficacy is manifested in multiple aspects: clinical studies have confirmed that pioglitazone (the standard therapeutic dose is 15–30 mg/day, which can be titrated up to a maximum of 45 mg/day according to the degree of IR and patient tolerability). This drug has been approved for marketing and is a classic first-line agent for the clinical treatment of MASLD complicated with T2DM (Dorkhan and Frid, 2007). Significantly improves hepatic histopathological features in patients with MASH, including the attenuation of hepatic steatosis, ballooning necrosis and lobular inflammation. A 36-month randomized controlled trial showed that more than 50% of liver biopsy-proven MASH patients achieved partial or complete resolution of steatohepatitis after treatment, with a sustained reduction in hepatic triglyceride levels (Pereira et al., 2025). Meanwhile, it enhances systemic insulin sensitivity, improves the homeostasis model assessment of insulin resistance (HOMA-IR) in MASLD patients, reduces hepatic gluconeogenesis, and alleviates the metabolic stress on the liver caused by blood glucose fluctuations, making it particularly suitable for MASLD patients with T2DM or prediabetes. However, there are conflicting findings regarding its effect on hepatic fibrosis: several meta-analyses have demonstrated no significant improvement in moderate to severe hepatic fibrosis (Boettcher et al., 2012), while subgroup analyses of patients with early-stage fibrosis (F1–F2 stages) revealed a mild reduction in fibrosis scores after 18 months of treatment, suggesting that the efficacy is associated with the baseline fibrosis severity of patients. Additional studies have indicated that pioglitazone may exert a potential ameliorative effect on early-stage fibrosis by inhibiting hepatic stellate cell activation and reducing collagen deposition (Musso et al., 2017). Pioglitazone improves lipid metabolism and insulin resistance by activating the PPARγ pathway. Activation of this pathway significantly upregulates serum APN expression and reduces the resistin/adiponectin ratio. Meanwhile, it inhibits hepatic stellate cell activation, thereby decreasing tissue inhibitor of metalloproteinase-1 (TIMP-1) expression and attenuating abnormal elevations in liver fibrosis-related biomarkers. Furthermore, PPARγ activation downregulates GPNMB expression in liver tissue, alleviates hepatic inflammation, and reduces serum miR-122 levels, reflecting the improvement of hepatocellular injury. In terms of combination therapy and safety, monotherapy with pioglitazone is prone to induce weight gain (a mean increase of 2–4 kg), and this adverse effect can be effectively counteracted by combination with sodium-glucose cotransporter 2 (SGLT-2) inhibitors (e.g., tofogliflozin) or metformin (Isaacs et al., 2025). Clinical data have shown that the incidence of weight gain is reduced by 40% in the metformin combination group compared with the monotherapy group, with a significant decrease in the risk of cardiovascular diseases. Beyond weight gain, pioglitazone may also increase the risk of fractures in elderly women, induce edema and heart failure; long-term use requires monitoring of bone mineral density and cardiac function, and the drug is contraindicated in patients with a history of bladder cancer (Viscoli et al., 2017). Additionally, there are differences in efficacy among special populations: female MASLD patients with glucose metabolism disorders who received pioglitazone combined with lifestyle interventions had a greater reduction in liver enzyme levels and a higher resolution rate of steatohepatitis than male patients, which is presumed to be associated with the regulatory effect of sex hormones on PPARγ.

### 3.2 Biguanides

#### 3.2.1 Core drug: metformin

Metformin exerts its pharmacological effects by inhibiting hepatic gluconeogenesis, reducing intestinal glucose absorption and enhancing insulin sensitivity in peripheral tissues. As a first-line agent for the treatment of T2DM, it also holds important adjunctive value in the management of MASLD (Ruan et al., 2023). This drug has been approved for marketing. It is a first-line agent for T2DM and a recommended adjunctive therapy for MASLD complicated with glucose metabolism disorders. Its clinical efficacy and application characteristics are manifested in multiple aspects, multiple randomized controlled trials have indicated that metformin (the standard initial adult dose is 500 mg per dose, twice daily, taken with meals; the dose can be adjusted according to blood glucose levels and tolerability after 1–2 weeks, with the maximum maintenance dose not exceeding 2550 mg daily, administered in 2–3 divided oral doses) fails to improve hepatic histopathological features (e.g., steatosis, inflammation and fibrosis staging) in MASLD patients, but it can significantly reduce serum transaminase levels with a mean decrease of 20%–30% (European Association for the Study of the LiverEuropean Association for the Study of Diabetes EASDEuropean Association for the S tudy of Obesity EASO, 2024). Meanwhile, its weight-loss effect (especially for abdominal obesity) and improvement in IR can indirectly reduce hepatic lipid deposition and lower the risk of HCC, exerting a positive impact on the long-term prognosis of MASLD patients with T2DM (Yasmin et al., 2021). However, the treatment of nonalcoholic fatty liver disease in children (TONIC) randomized controlled trial in pediatric and adolescent MASLD patients showed no significant differences in transaminase reduction and hepatic histopathological improvement compared with placebo, suggesting that its efficacy may be age-dependent, with more definite benefits observed in adult patients. In terms of combination therapy, the combined use of metformin with traditional Chinese medicine (TCM) compound formulas has become a research hotspot. A clinical study involving 126 MASLD patients with the syndrome of damp-heat accumulation showed that after 12 weeks of treatment with metformin combined with Danzhi Tiaozhi Decoction (composed of *Bupleuri Radix*, *Coptidis Rhizoma*, *Salviae Miltiorrhizae Radix* et Rhizoma, etc.), the improvements in glucose metabolism indices (FPG, Glycated hemoglobin A1c [HbA1c]), lipid metabolism indices TG, low-density lipoprotein cholesterol (LDL-C) and liver enzyme levels in patients were significantly superior to those in the metformin monotherapy group, with the total effective rate of TCM syndrome reaching 88.89% (Qu et al., 2025), and no increase in the incidence of adverse reactions. This combination regimen achieves multi-target intervention for MASLD through the synergistic effects of Western medicine in regulating metabolism and TCM in clearing heat, eliminating dampness and soothing the liver (Zambran et al., 2025). Metformin enhances insulin sensitivity and inhibits hepatic gluconeogenesis by activating the AMPK pathway. Activation of this pathway reduces serum levels of branched-chain amino acids (BCAAs) and the leucine/isoleucine ratio, thereby ameliorating abnormalities in metabolic biomarkers. Concurrently, AMPK activation moderately upregulates serum adiponectin levels and downregulates hepatic expression of SREBP1c, leading to decreased levels of lipid synthesis-related biomarkers such as diacylglycerols (DAGs), which indirectly reflects the alleviation of hepatic lipid accumulation. Regarding safety, the common adverse reactions of metformin are gastrointestinal symptoms (diarrhea, nausea), which mostly occur at the initial stage of medication and can be gradually tolerated with the prolongation of treatment course. Long-term use may lead to vitamin B12 deficiency, thus regular monitoring of serum vitamin B12 levels is required. Metformin is contraindicated in patients with severe renal insufficiency, severe infection and metabolic acidosis (Tao et al., 2024).

### 3.3 SGLT-2 inhibitors

#### 3.3.1 Representative drugs: empagliflozin, dapagliflozin, lificogliptin

SGLT-2 inhibitors are a class of agents that exert their effects by blocking glucose reabsorption in the proximal renal tubule and promoting glycosuria, with concomitant weight loss, blood pressure-lowering and cardiorenal protective effects. Their therapeutic value in MASLD stems from the dual actions of metabolic improvement and direct hepatic protection (Zhang and Hu, 2025). In terms of clinical efficacy, empagliflozin (the standard initial adult dose is 10 mg once daily, administered in the morning; the dose may be titrated to a maintenance dose of 25 mg once daily after 2 weeks according to glycemic control and tolerability) achieved a 67% rate of hepatic fat regression in MASLD patients after 24 weeks of treatment at 25 mg once daily, which was significantly higher than the 26% rate in the placebo group. Meanwhile, the improvement rates of hepatocellular ballooning and liver fibrosis reached 78% and 44%, respectively, with statistically significant differences in all indicators (*P* < 0.05) (Kahl et al., 2020). This drug has been approved for marketing, and its indication for MASLD treatment has been recommended in multiple clinical guidelines. For dapagliflozin (the standard initial adult dose is 5 mg once daily, administered in the morning; the maximum maintenance dose of 10 mg once daily may be achieved after 1–2 weeks based on glycemic status and tolerability), multiple randomized controlled trials spanning 8–52 weeks have confirmed its ability to improve non-invasive indices of steatosis and fibrosis such as magnetic resonance proton density fat fraction (MRI-PDFF) and liver stiffness values, though direct histopathological evidence of improvement still requires validation in large-sample studies (Cada et al., 2014). This drug has been approved for marketing and is widely used clinically in MASLD patients complicated with type 2 diabetes/obesity. As a dual SGLT-1/SGLT-2 inhibitor, lificogliptin regulates glucose metabolism through both intestinal and renal pathways, exhibiting unique advantages. At a maintenance dose of 150 mg once daily (the standard initial adult dose is 50 mg once daily, administered in the morning; the dose may be adjusted to 150 mg once daily after 2 weeks according to glycemic control), lificogliptin reduced placebo-corrected alanine aminotransferase (ALT) levels by 22% and hepatic fat content by 29% in MASLD patients, with two-thirds of patients achieving a relative reduction of ≥30% in hepatic fat content. This agent is thus particularly suitable for MASLD patients complicated with hyperlipidemia (Tian et al., 2023). This drug has been approved for marketing and is widely used clinically in MASLD patients complicated with type 2 diabetes/obesity. In addition, the class-specific weight loss of 3–5 kg on average and blood pressure-lowering effects of SGLT-2 inhibitors can further reduce the metabolic burden of MASLD patients and decrease the risk of cardiovascular complications, which are highly consistent with the multisystem pathological characteristics of MASLD (Wu Q. et al., 2025). SGLT-2 inhibitors indirectly improve hepatic lipid metabolism by inhibiting renal glucose reabsorption, while directly activating the hepatic PPARα pathway to promote fatty acid oxidation. Activation of this pathway reduces serum ceramide levels and attenuates abnormalities in lipotoxicity-related biomarkers. In addition, these drugs decrease serum soluble lectin-like oxidized low-density lipoprotein receptor-1 (sLOX-1) levels and alleviate oxidative stress injury in hepatocytes, leading to concurrent reductions in imaging biomarkers such as MRI-PDFF and enzymatic biomarkers including serum ALT and AST, which reflect improvements in hepatic steatosis and inflammation. Regarding safety, SGLT-2 inhibitors are associated with a risk of common genitourinary tract infections and dehydration, while the incidence of rare adverse reactions such as diabetic ketoacidosis and lower-extremity amputation is less than 0.1%. Sufficient water intake should be ensured during treatment, with close monitoring of blood glucose and renal function. These agents are mainly indicated for MASLD patients complicated with type 2 diabetes or obesity, and are contraindicated in patients with a hypersensitivity to SGLT-2 inhibitors or severe renal insufficiency (Bhanushali et al., 2024).

### 3.4 GLP-1 receptor agonists

#### 3.4.1 Representative drugs: liraglutide, semaglutide

GLP-1 receptor agonists act by mimicking endogenous GLP-1 to promote insulin secretion and inhibit glucagon release, and exhibit marked weight-loss effects as well as delayed gastric emptying. As an important emerging class of agents for MASLD treatment, their representative drugs are liraglutide and semaglutide (Singh et al., 2024). In terms of clinical efficacy, MRI-PDFF assessments demonstrated that after 48 weeks of treatment with liraglutide at a maintenance dose of 1.8 mg once daily via subcutaneous injection (the standard initial adult dose is 0.6 mg once daily subcutaneously, titrated upward by 0.6 mg weekly to a maintenance dose of 1.8 mg once daily, with a maximum dose not exceeding 1.8 mg daily), MASLD patients achieved a mean 40% reduction in hepatic fat content, with 39% of patients experiencing MASH resolution and no worsening of liver fibrosis (Armstrong et al., 2016). This drug has been approved for marketing and is an important emerging agent for the treatment of MASLD/MASH. For semaglutide (for the treatment of metabolic disorders in adults, the standard initial dose is 0.25 mg once weekly via subcutaneous injection for 4 weeks, then adjusted to 0.5 mg once weekly; the dose may be further titrated to a maintenance dose of 1.0 mg once weekly after 8 weeks based on efficacy and tolerability), subcutaneous administration in phase II clinical trials significantly reduced hepatic inflammatory cell infiltration and hepatocellular degeneration, and effectively blocked the progression of liver fibrosis, though it showed no significant improvement in established fibrosis staging (Simon, 2025). This drug has been approved for marketing, and its long-acting formulation is one of the preferred agents for MASLD patients with obesity. This class of agents also exerts comprehensive metabolic-improving effects: semaglutide achieved a mean 15% weight loss after 1 year of treatment, and simultaneously effectively controlled blood glucose levels and reduced TG and LDL-C concentrations. By alleviating the metabolic drivers of MASLD through multiple dimensions, GLP-1 receptor agonists are particularly suitable for MASLD patients complicated with obesity and type 2 diabetes (Zasadzińska and Borowski, 2025). GLP-1 receptor agonists suppress appetite and improve glycolipid metabolism by activating the GLP-1 pathway. They simultaneously upregulate adiponectin secretion in adipose tissue and activate the hepatic AMPK/PPARα pathway, leading to significantly elevated serum adiponectin levels, decreased resistin levels, and a reduced resistin/adiponectin ratio (Chrysavgis et al., 2025). Furthermore, these drugs inhibit the hepatic nuclear factor kappa-B (NF-κB) pathway and reduce serum levels of inflammatory biomarkers such as TNF-α and IL-6. They also downregulate hepatic GPNMB expression, attenuate M2 macrophage-related inflammatory injury, and decrease serum miR-34a levels, reflecting the alleviation of hepatic inflammation (Ren et al., 2025). In terms of safety and tolerability, the predominant adverse reactions of these agents are gastrointestinal symptoms, including nausea, vomiting and diarrhea, with an incidence of approximately 30%–40%. These reactions are mostly mild to moderate, tend to alleviate gradually with prolonged treatment, and are dose-dependent; initiating treatment at a low dose can reduce the incidence of such adverse effects (Weiskirchen, 2025). A key special precaution is that these drugs are contraindicated in patients with a personal or family history of medullary thyroid carcinoma. For long-term use, regular monitoring of thyroid function and pancreatitis-related biomarkers (e.g., amylase, lipase) is required to ensure medication safety (Mittal et al., 2025).

### 3.5 Dual/triple receptor agonists

#### 3.5.1 Dual receptor agonist: tirzepatide (a GIP/GLP-1 dual agonist)

As a novel GIP/GLP-1 dual receptor agonist, tirzepatide exerts synergistic hypoglycemic, weight-loss and hepatoprotective effects by simultaneously activating the glucose-dependent insulinotropic polypeptide (GIP) receptor and the GLP-1 receptor. Its comprehensive efficacy is significantly superior to that of single GLP-1 receptor agonists, providing a novel multi-target intervention strategy for the treatment of MASLD (Nowak et al., 2025). In terms of clinical efficacy, results from the phase II clinical trial demonstrated that subcutaneous administration of tirzepatide (the standard initial adult dose is 2.5 mg once weekly via subcutaneous injection, titrated upward by 2.5 mg every 4 weeks to a target maintenance dose of 10 mg or 15 mg once weekly, with a maximum dose not exceeding 15 mg once weekly) exhibited a definite ameliorative effect on liver-related indices, after 52 weeks of treatment in the 10 mg and 15 mg dose groups, serum ALT levels were significantly reduced in MASLD patients with a decrease, and aspartate aminotransferase (AST) levels were markedly decreased in all groups except, suggesting effective alleviation of hepatocellular injury (Loomba et al., 2024). After 52 weeks of treatment, MRI-PDFF assessments confirmed a reduction of more than 30% in hepatic fat content from baseline in patients, and the significant improvement in lipid deposition provided critical support for blocking disease progression (Targher, 2022). This drug is currently in the late-stage clinical (Phase III) trial, and its indication for MASLD/MASH treatment has not yet been officially approved, while clinical data have demonstrated significant hepatoprotective effects. Although direct evidence of hepatic histopathological improvement (e.g., resolution of inflammation, reversal of fibrosis) has not yet been obtained, its potent regulatory effects on glucose and lipid metabolism—including a significant reduction in glycated hemoglobin, decreased visceral fat accumulation, and improved IR—indirectly suggest its potential therapeutic value for MASH, making it particularly suitable for MASLD patients complicated with type 2 diabetes or obesity (Arai et al., 2025). Tirzepatide improves glycolipid metabolism and hepatic lipid accumulation by co-activating the GIP/GLP-1 pathway (Hu et al., 2025). Activation of this pathway significantly reduces serum levels of metabolic biomarkers including glycated HbA1c and TG, while upregulating serum adiponectin levels and downregulating hepatic expression of SREBP1c and ChREBP. This results in decreased levels of hepatic lipogenesis-related biomarkers such as DAGs and ceramides. In addition, tirzepatide lowers serum TIMP-1 levels and attenuates abnormal elevations in liver fibrosis-related biomarkers (Liang et al., 2025). In terms of safety, the adverse reaction profile of tirzepatide is similar to that of GLP-1 receptor agonists, predominantly presenting with mild gastrointestinal symptoms such as nausea, diarrhea and vomiting. Most of these reactions are transient, can be gradually tolerated with prolonged treatment, and no new safety risks have been identified, indicating a good overall tolerability (Tong et al., 2023). Currently, in-depth research on its hepatic histopathological ameliorative effect is still underway. Future phase III clinical trials with larger sample sizes are required to further validate its direct therapeutic value for MASH, thereby providing more robust evidence to support its extensive application in the treatment of MASLD (Zafer et al., 2025).

#### 3.5.2 Triple receptor agonist: retatrutide (a GIP/GLP-1/GCGR triple agonist)

As the world’s first GIP/GLP-1/GCGR (glucagon receptor) triple receptor agonist, retatrutide establishes a multi-target synergistic regulatory network by simultaneously activating the GIP receptor, GLP-1 receptor and GCGR. On the one hand, it enhances insulin secretion and inhibits glucagon release to optimize glucose metabolism; on the other hand, it potentiates lipolysis and energy expenditure and suppresses appetite to achieve weight loss. Meanwhile, it reduces hepatic lipid deposition by regulating hepatic lipid metabolic pathways, exerting a synergistic effect of hypoglycemia, weight loss and hepatoprotection in a comprehensive manner, with its potency significantly superior to that of single receptor agonists (Coskun et al., 2022). In terms of clinical efficacy, data from phase II clinical trials have demonstrated a breakthrough metabolic improvement effect of this agent, after 24 weeks of treatment with retatrutide at doses of 8 mg or 12 mg (the recommended initial dose in adult clinical studies is 1 mg once weekly via subcutaneous injection, titrated upward by 2 mg every 4 weeks according to patient tolerability and efficacy to a target maintenance dose of 8 mg or 12 mg once weekly; the maximum exploratory dose in current clinical studies is 12 mg once weekly), 90% of MASLD patients achieved disease resolution. MRI-PDFF assessments showed that hepatic fat content was reduced by 81.4% and 82.4% in the respective dose groups, compared with only a 0.2% reduction in the placebo group, with a statistically significant intergroup difference (P < 0.001) (Sanyal et al., 2024). When the treatment duration was extended to 48 weeks, the reduction in hepatic fat content was further enhanced, reaching 81.7% in the 8 mg group and as high as 86% in the 12 mg group, which far exceeded the efficacy of currently used clinical agents. At the same time, MASLD-related metabolic risk factors in patients, including waist circumference, systolic and diastolic blood pressure, glycated HbA1c and TG, were all significantly improved, reducing the risk of disease progression through multiple dimensions (Sanyal et al., 2023). This drug is currently in the mid-stage clinical (Phase II) trial, as a promising pipeline agent for MASLD treatment, with no approved indications to date. As one of the most potent agents for weight loss and metabolic improvement to date, retatrutide provides a novel therapeutic option for patients with severe obesity complicated by MASLD, and is particularly suitable for those with poor response to conventional drugs or combined multiple metabolic abnormalities. However, several key issues need to be addressed for its clinical application: to date, there is no direct evidence demonstrating its ameliorative effect on hepatic fibrosis, and the relevant efficacy still requires further verification by gold standards such as liver biopsy in phase III clinical trials (Sinha and Ghosal, 2025). Retatrutide achieves coordinated regulation of glycolipid metabolism and energy expenditure by activating the GIP/GLP-1/GCGR triple pathway. Activation of this pathway gradually normalizes serum fibroblast growth factor 21 (FGF21) levels from a compensatory increase, reflecting an improvement in hepatic FGF21 resistance. Meanwhile, retatrutide significantly reduces serum levels of GPNMB and sLOX-1, alleviating hepatic inflammation and oxidative stress. It decreases liver fat content–related biomarkers (MRI-PDFF) by more than 80% (Pasqualotto et al., 2024), and simultaneously improves serum levels of branched-chain amino acids and bile acid metabolism markers (e.g., LCA). These changes collectively reflect the multipathway regulatory effects of the drug. The potential long-term effects on the cardiovascular system, safety regarding bone metabolism, and tolerability in special populations (e.g., patients with complicated cardiovascular diseases or osteoporosis) also await supporting data from studies with larger sample sizes and longer follow-up periods. With the advancement of in-depth clinical research in the future, retatrutide is expected to become a core therapeutic agent for MASLD patients complicated with obesity and type 2 diabetes, providing a new breakthrough for the comprehensive management of metabolic disorders.

All the aforementioned therapeutic agents for MASLD act by targeting and modulating core pathological pathways, such as hepatic lipid metabolism, insulin resistance, and immune inflammation. The activation or inhibition of distinct pathways by these pharmacologic interventions directly translates into altered expression levels of MASLD-specific, metabolic, and epigenetic biomarkers. The following section systematically delineates the core mechanistic pathways underlying MASLD pharmacotherapy and defines the associations between individual pathways and clinical agents, thereby establishing a framework for analyses of drug–pathway–biomarker subsequent interrelationships (Table 1).

**TABLE 1:** Medications for metabolic-associated steatotic liver disease.

Drug class	Drug name	Usual adult dosage	Key therapeutic characteristics	Major safety precautions/Key safety considerations
Thiazolidinediones	Pioglitazone	Initiate at 15–30 mg/day; may be titrated to a maximum of 45 mg/day based on the degree of insulin resistance and tolerability	Improves hepatic histology in patients with MASH; ameliorates insulin resistance; has potential beneficial effects on early hepatic fibrosis	Weight gain; increased fracture risk in elderly women; edema and heart failure; contraindicated in patients with a history of bladder cancer
Biguanides	Metformin	Initiate at 500 mg twice daily with meals; titrate after 1–2 weeks. Maximum maintenance dose does not exceed 2550 mg/day, administered in 2–3 divided doses	Reduces serum transaminase levels; promotes weight loss and improves insulin resistance; indirectly decreases hepatic fat accumulation	Gastrointestinal reactions; long-term use may cause vitamin B12 deficiency; contraindicated in patients with severe renal impairment, infection, or metabolic acidosis
SGLT-2 inhibitors	Empagliflozin	Initiate at 10 mg once daily in the morning; may be titrated to a maintenance dose of 25 mg/day after 2 weeks	Hepatic fat resolution rate: 67%; improves hepatocellular ballooning and fibrosis; provides weight reduction, blood pressure lowering, and cardiorenal protection	Risk of genitourinary tract infections and dehydration; rare cases of ketoacidosis and lower limb amputation; contraindicated in patients with severe renal impairment
SGLT-2 inhibitors	Dapagliflozin	Initiate at 5 mg once daily in the morning; may be titrated to a maximum maintenance dose of 10 mg/day after 1–2 weeks	Improves non-invasive markers including MRI-PDFF and liver stiffness; promotes weight loss and lowers blood pressure	Risk of genitourinary tract infections and dehydration; rare cases of ketoacidosis and lower limb amputation; contraindicated in patients with severe renal impairment
Dual SGLT-1/SGLT-2 inhibitors	Lificogliptin	Initiate at 50 mg once daily in the morning; may be titrated to a maintenance dose of 150 mg/day after 2 weeks	MASLD patientsReduces ALT levels by 22% and hepatic fat content by 29%; suitable for MASLD patients with hyperlipidemia	Risk of genitourinary tract infections and dehydration; rare cases of ketoacidosis and lower limb amputation; contraindicated in patients with severe renal impairment
GLP-1 receptor agonists	Lificogliptin	Initiate at 0.6 mg subcutaneously once daily; titrate by 0.6 mg weekly to a maintenance dose of 1.8 mg/day	Reduces hepatic fat content by 40%; 39% of patients achieve MASH resolution; promotes weight loss and glycemic control	Gastrointestinal reactions; contraindicated in patients with a personal or family history of medullary thyroid carcinoma; monitor thyroid function and markers of pancreatitis
GLP-1 receptor agonists	Semaglutide	Initiate at 0.25 mg subcutaneously once weekly; titrate to 0.5 mg after 4 weeks, and may be increased to 1.0 mg after 8 weeks	Reduces hepatic inflammatory cell infiltration and hepatocellular degeneration; halts fibrosis progression; induces 15% weight loss and comprehensively improves metabolic parameters	Gastrointestinal reactions; contraindicated in patients with a personal or family history of medullary thyroid carcinoma; monitor thyroid function and markers of pancreatitis
GIP/GLP-1 dual receptor agonists	Tirzepatide	Initiate at 2.5 mg subcutaneously once weekly; titrate by 2.5 mg every 4 weeks to a maintenance dose of 10–15 mg per injection	ALT and AST are significantly reduced in most groups; hepatic fat content decreases by ≥ 30%; potently improves insulin resistance and reduces visceral fat	Mild gastrointestinal reactions; overall good tolerability
GIP/GLP-1/GCGR triple receptor agonists	Retatrutide	Initiate at 1 mg subcutaneously once weekly; titrate by 2 mg every 4 weeks to a maintenance dose of 8–12 mg per injection	90% of patients achieve disease resolution; hepatic fat content decreases by more than 80%; significantly improves metabolic parameters including waist circumference, blood pressure, and blood lipids	Long-term safety has not been established; attention should be paid to cardiovascular and bone metabolism effects; awaiting validation from phase III clinical data
Thyroid hormone receptor β agonist	Resmetirom	Initial dose: 80 mg once daily orally, which may be adjusted to 100 mg once daily based on efficacy and tolerability	59% of patients with MASH achieved disease resolution without fibrosis worsening; hepatic fat content decreased by 40%–45%; improved lipid metabolism and reduced cardiovascular risk	No significant adverse effects on cardiovascular or bone metabolism; occasional mild gastrointestinal reactions (nausea, diarrhea) were generally well-tolerated; clinical trial status: Phase III (late stage), not yet approved

### 3.6 Resmetirom

#### 3.6.1 Resmetirom: thyroid hormone receptor β (THRβ) agonist

Resmetirom is a highly selective THRβ agonist that exerts targeted regulatory effects on hepatic lipid metabolism and inflammatory responses. As a liver-targeted agent, it exhibits minimal binding to thyroid hormone receptor α (THRα), thereby avoiding adverse effects such as cardiovascular and skeletal abnormalities caused by non-specific activation of THRα. It has demonstrated favorable safety profiles in clinical studies and represents a promising candidate for the treatment of metabolic-associated steatohepatitis (MASH). Currently, resmetirom is in the late clinical (III) stage, and has not yet been approved for marketing, with definitive efficacy confirmed in global multicenter Phase III trials. The core mechanism by which resmetirom improves MASLD/MASH is mediated through the specific activation of hepatic THRβ. On the one hand, it regulates lipid metabolism: it directly upregulates the expression of genes involved in hepatic fatty acid β-oxidation and VLDL assembly/secretion, markedly promoting fatty acid catabolism and hepatic TG export. Meanwhile, it downregulates the expression of the central DNL transcription factors SREBP1c and ChREBP, thereby inhibiting hepatic fatty acid and TG synthesis. This dual mechanism—reducing synthesis while enhancing breakdown and export—reverses hepatic lipid accumulation and has been validated in both preclinical animal studies and human clinical trials (Harrison et al., 2023). On the other hand, resmetirom inhibits pathological processes associated with steatohepatitis: via THRβ activation, it suppresses hepatic NF-κB pathway activation, reduces the expression and secretion of pro-inflammatory cytokines in liver macrophages, and alleviates lobular inflammation and hepatocellular ballooning. It also inhibits hepatic stellate cell activation, decreases the expression of fibrosis-related biomarkers, and delays the progression of liver fibrosis in patients with MASH (Raja et al., 2024). Clinical trials of resmetirom for MASH have advanced to late stages. Phase II results showed that, in MASH patients with fibrosis, the proportion achieving disease resolution without fibrosis worsening was significantly higher in the resmetirom group than in the placebo group, accompanied by substantial reductions in liver fat content and marked improvements in serum transaminase levels. Global multicenter phase III trials further confirmed its efficacy: in the intention-to-treat population, the MASH resolution rate was significantly superior in the resmetirom group compared with placebo. The drug also effectively improved lipid metabolic parameters without significant adverse effects on heart rate, blood pressure, or bone metabolism (Dutta et al., 2024). With regard to lipid metabolism improvement, resmetirom significantly reduces hepatic TG content and serum lipid levels in MASLD patients, helping to ameliorate systemic dyslipidemia and lower the risk of cardiovascular complications. Beyond reversing hepatic steatosis, it also reduces histological scores for lobular inflammation and hepatocellular ballooning, inhibits oxidative stress, decreases hepatocyte apoptosis, and delays liver fibrosis by suppressing hepatic stellate cell activation, with particularly notable benefits in patients with early-stage fibrosis (Liao et al., 2022).

Besides resmetirom, other promising pipeline drugs for MASLD have entered advanced clinical research stages and shown notable therapeutic potential, among which Aramchol and Aldafermin stand out with their unique targeted mechanisms. Aramchol is a highly selective stearoyl-CoA desaturase 1 (SCD1) inhibitor currently in Phase 3 clinical trials, targeting the core lipid metabolism disorder of MASLD. As a key rate-limiting enzyme in hepatic *de novo* lipogenesis, SCD1 is abnormally upregulated in MASLD patients, accelerating the synthesis of monounsaturated fatty acids and promoting triglyceride and ceramide accumulation in hepatocytes, which in turn triggers steatosis and subsequent inflammation and fibrosis (Syed-Abdul, 2023). By specifically inhibiting hepatic SCD1 activity, Aramchol directly blocks the abnormal lipogenesis process, reverses hepatic lipid deposition, and synergistically downregulates the expression of lipid synthesis-related transcription factors such as SREBP1c, forming a targeted intervention for lipid metabolic dysfunction. Aramchol is a highly selective SCD1 inhibitor currently in late clinical (III) stage, and Aldafermin, a recombinant analog of FGF19 in mid-stage clinical (IIB) stage, exerts its hepatoprotective effects by regulating the gut-liver axis, a critical pathological link in MASLD development. It mimics the biological activity of endogenous FGF19 to bind to the hepatic FGFR4/β-klotho complex, which not only inhibits CYP7A1, the key enzyme of bile acid synthesis, to reduce bile acid-induced hepatic inflammatory injury, but also inhibits hepatic lipogenesis and stellate cell activation, thus simultaneously ameliorating hepatic steatosis, inflammation and early fibrosis (Kurosu et al., 2007). Both drugs have demonstrated good tolerability in early clinical trials, with no serious adverse reactions reported, and their targeted regulatory effects on different pathological pathways of MASLD further enrich the research and development landscape of specific therapeutic drugs for this disease, bringing new hopes for clinical precision treatment (Rinella et al., 2024).

## 4 Mechanistic pathways of therapeutic agents for MASLD

The development and progression of MASLD are closely associated with hepatic lipid metabolism disorder, mitochondrial dysfunction, impaired lipid transport and an imbalanced immune-inflammatory microenvironment. Its therapeutic agents and interventions exert their effects primarily through two core approaches: precise regulation via targeted pathways in Western medicine and synergistic modulation via multi-targets in TCM, ultimately achieving the goals of ameliorating hepatic lipid accumulation, restoring the normal physiological functions of the liver, and delaying or reversing hepatic fibrosis (Schwärzle et al., 2024). The initiation and progression of MASLD are closely associated with dysregulated pathways including hepatic lipid synthesis, fatty acid oxidation, lipid transport, and immunometabolic disorders. All clinical and investigational drugs exert precise regulatory effects by targeting these core pathways. The modulatory effects of drugs on these pathways can be quantitatively evaluated by measuring changes in the expression of biomarkers such as GPNMB, adiponectin, and miR-122 (Tobaruela-Resola et al., 2025), thereby forming an integrated regulatory network of drug intervention–pathway modulation–biomarker response. This section will dissect the regulatory mechanisms of each core pathway and clarify the targeted modes of action of clinical drugs on individual pathways.

### 4.1 Targeting the lipid synthesis pathway

#### 4.1.1 SREBP1c pathway inhibitors

**FIGURE 1:**
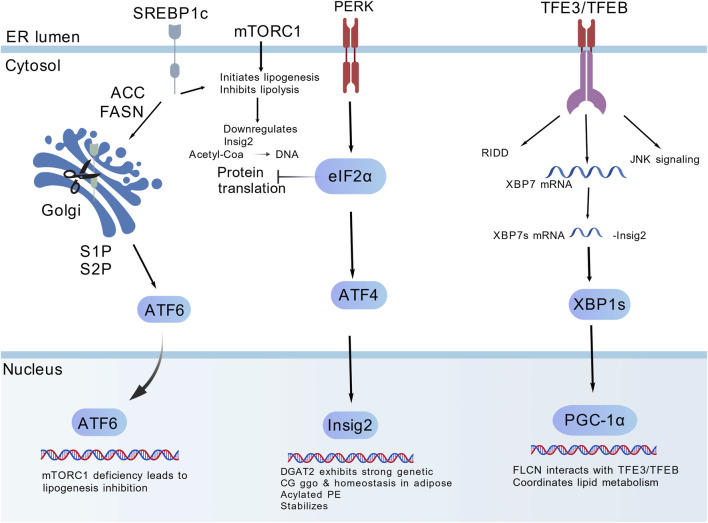
Mechanism diagram of SREBP1c pathway inhibitors regulating hepatic DNL.

SREBP1c is a core transcription factor regulating DNL, and its activation is dependent on upstream signaling pathways and its own proteolytic maturation (Sanders and Griffin, 2016). Under physiological conditions, SREBP1c is anchored to the endoplasmic reticulum membrane as an inactive precursor; when cellular lipid levels decrease or insulin signaling is activated, the precursor is hydrolyzed into active fragments that translocate to the nucleus, bind to the promoters of genes such as ACC and FASN, and initiate DNL (Ferré et al., 2021). mTORC1 serves as a core upstream regulatory factor that promotes SREBP1c activation via phosphorylation; its inhibitors can activate the TFE3/TFEB-Insig2 pathway, enabling Insig2 to form a complex with the SREBP1c precursor and Scap, anchor the former to the endoplasmic reticulum, block proteolytic activation, and reduce hepatic TG synthesis and accumulation. As a key terminal enzyme in TG synthesis, DGAT2 inhibitors can directly inhibit TG synthesis, as well as increase the content of phosphatidylethanolamine in the endoplasmic reticulum, enhance the stability of the Insig2-SREBP1c-Scap complex, and exert a dual inhibitory effect on pathway activity (Gosis et al., 2022). In addition, hepatocyte-specific FLCN deletion can enhance the nuclear localization of TFE3/TFEB, upregulate Insig2 expression, inhibit SREBP1c hydrolysis, block DNL, and reduce fatty acid synthesis and TG deposition in hepatocytes (Paquette et al., 2021) (Figure 1).

This pathway represents one of the core targeted pathways for drugs including pioglitazone, tirzepatide, and retatrutide. Pioglitazone indirectly inhibits the activation of SREBP1c via the PPARγ pathway, whereas tirzepatide and retatrutide downregulate SREBP1c expression through the GIP/GLP-1 pathway. Inhibition of this pathway by these agents reduces hepatic lipogenesis-related biomarkers (DAGs and ceramides), reflecting the efficacy of pathway regulation.

#### 4.1.2 ChREBP pathway modulators

**FIGURE 2:**
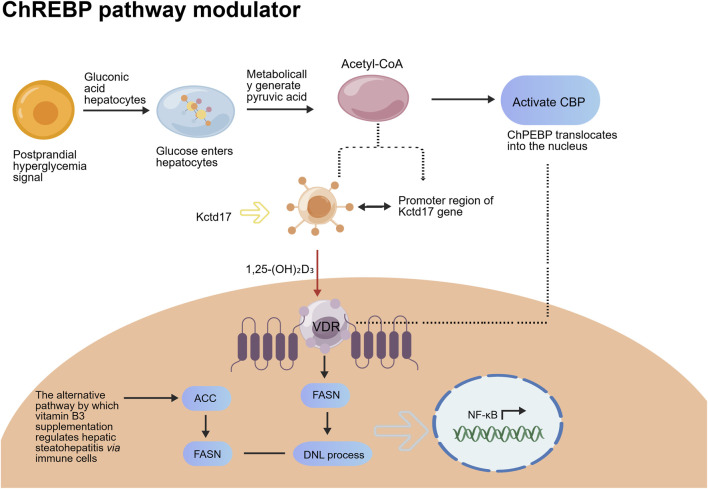
Mechanism diagram of vitamin D3 regulating ChREBP-mediated hepatic DNL pathway.

ChREBP is a key transcription factor regulating the conversion of glucose to fatty acids, which primarily responds to postprandial hyperglycemia signals (Linden et al., 2018). When postprandial blood glucose levels rise, glucose enters hepatocytes and is metabolized to pyruvate, which is further converted to acetyl-CoA. Acetyl-CoA can activate ChREBP activity, enabling its nuclear translocation to initiate the expression of downstream fatty acid synthesis genes such as ACC and FASN, thereby promoting the conversion of glucose to fatty acids and exacerbating the progression of DNL (Iizuka, 2013). Kctd17 (potassium channel tetramerization domain-containing protein 17) is a critical upstream regulatory factor of ChREBP, which can enhance ChREBP protein stability and activity through binding to ChREBP. Vitamin D3 exerts its effects via its active metabolite 1,25-(OH)_2_D_3_: this metabolite binds to the vitamin D receptor in hepatocytes to form a complex, which then binds to the promoter region of the Kctd17 gene and represses its transcriptional expression (Oh et al., 2023). Downregulated Kctd17 expression impairs its binding capacity to ChREBP, leading to decreased ChREBP protein stability and activity, which abrogates the effective initiation of downstream fatty acid synthesis gene expression. Consequently, this reduces the postprandial hyperglycemia-induced conversion of glucose to fatty acids, blocks the ChREBP-mediated DNL pathway, and alleviates hepatic lipid accumulation. In addition, vitamin D3 can indirectly attenuate hepatic inflammation by regulating immune cell function, thereby exerting an adjuvant effect on ameliorating the pathological state of MASLD (Shymanskyi et al., 2025) (Figure 2).

Itamin D3 serves as a classic modulator of this pathway. Clinical hypoglycemic agents such as metformin and SGLT-2 inhibitors can indirectly suppress the activation of the ChREBP pathway by improving glucose metabolism, thereby reducing the conversion of glucose to fatty acids. Regulation of this pathway decreases serum branched-chain amino acid levels and ameliorates abnormalities in metabolic biomarkers.

### 4.2 Improving the fatty acid oxidation pathway

Impaired mitochondrial fatty acid β-oxidation in hepatocytes represents another core pathological feature of MASLD, which leads to the abnormal accumulation of free fatty acids (FFAs) in hepatocytes and further exacerbates lipid deposition and oxidative stress. The therapeutic core of this pathway is to activate key targets such as PPARα, restore mitochondrial fatty acid β-oxidation capacity, and promote the catabolism of FFAs (Theys et al., 2024).

**FIGURE 3:**
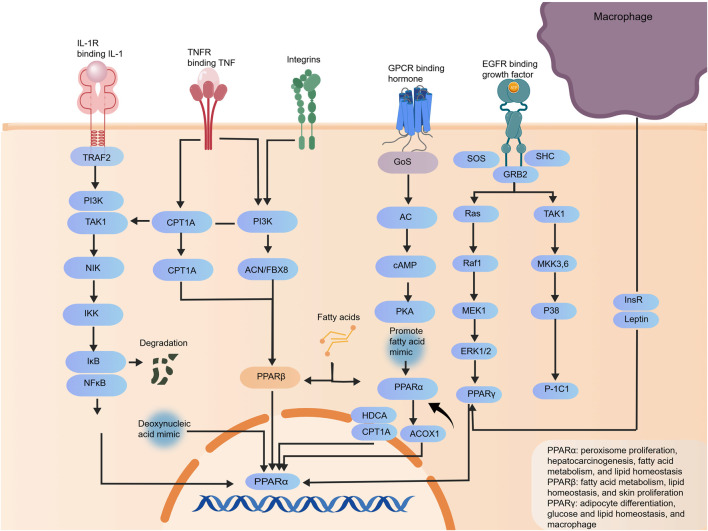
Mechanism diagram of PPARα pathway activation improving hepatocellular fatty acid β-oxidation.

PPARα is a core transcription factor regulating fatty acid oxidation, which is mainly expressed in metabolically active tissues such as the liver and skeletal muscle. Upon activation, it initiates the expression of downstream genes related to fatty acid oxidation (Fougerat et al., 2024). PPARα activity is significantly reduced in hepatocytes of MASLD patients, resulting in insufficient expression of key enzymes for fatty acid oxidation and impaired catabolism of FFAs. Hyodeoxycholic acid (HDCA) can enhance PPARα activity by regulating its subcellular localization: HDCA binds to bile acid receptors in hepatocytes and promotes the translocation of PPARα from the cytoplasm to the nucleus. Nuclear PPARα forms a heterodimer with the retinoid X receptor (RXR), which binds to the peroxisome proliferator-responsive element (PPRE) of downstream genes such as CPT1A and ACOX1 and initiates their transcription (Zhong et al., 2023). CPT1A facilitates the entry of FFAs into mitochondria, while ACOX1 assists in the catabolism of long-chain fatty acids; these two enzymes synergistically enhance fatty acid oxidation and reduce the accumulation of FFAs and TG in hepatocytes. The 5:2 intermittent fasting regimen (normal diet for 5 days and calorie restriction for 2 days per week) activates the PPARα pathway by modulating the metabolic microenvironment (Gallage et al., 2024), during the fasting period, reduced energy intake in the body triggers the mobilization and release of FFAs from adipose tissue, and FFAs act as endogenous ligands to bind and activate PPARα. The activated PPARα-RXR heterodimer initiates the expression of genes such as CYP4A10 and PCK1, CYP4A10 is involved in fatty acid ω-oxidation, and PCK1 improves lipid metabolism by regulating glucose metabolic homeostasis, enhances mitochondrial energy metabolism, restores the efficiency of fatty acid β-oxidation, and alleviates hepatic steatosis (Gallage et al., 2023). In addition, it can reduce oxidative stress products, mitigate mitochondrial damage, and further enhance fatty acid oxidation capacity (Figure 3).

PPARα serves as a core target for drugs including SGLT-2 inhibitors, liraglutide, and semaglutide (Zhou et al., 2025). SGLT-2 inhibitors directly activate the PPARα pathway to promote fatty acid oxidation, whereas GLP-1 receptor agonists indirectly activate this pathway by upregulating adiponectin. Activation of this pathway by these agents reduces serum ceramide levels, enhances the expression of the hepatic fatty acid oxidation–related enzyme CPT1A, and decreases serum miR-122 levels, reflecting the amelioration of hepatocellular injury (Antoniou et al., 2025).

**FIGURE 4:**
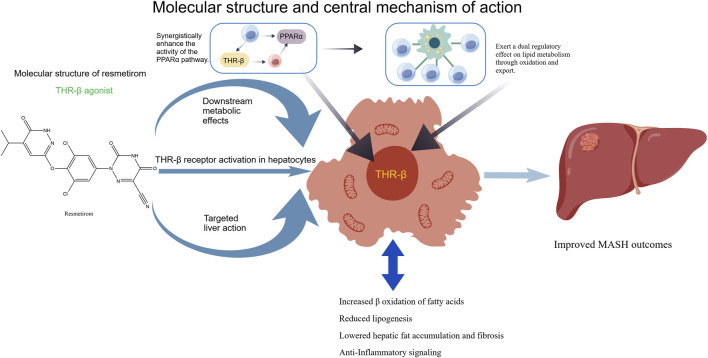
The molecular structure of resmetirom and its central mechanism of action against MASLD.

In addition, the thyroid hormone receptor *β* agonist resmetirom can directly activate THR*β* and synergistically enhance the activity of the PPARα pathway, further upregulating the expression of genes related to fatty acid *β*-oxidation while promoting VLDL-mediated hepatic lipid export, thereby forming a dual regulatory effect of lipid metabolism through “oxidation plus export” (Liu J. et al., 2025) (Figure 4).

### 4.3 Promoting the lipid transport pathway

TG synthesized in hepatocytes need to be assembled with apolipoprotein B (ApoB) and other proteins to VLDL. Hepatic TG is transported to peripheral tissues (e.g., adipose tissue, skeletal muscle) for metabolism via VLDL secretion. Impaired assembly or secretion of VLDL in hepatocytes of MASLD patients results in the failure of effective TG export from the liver, which further exacerbates hepatic lipid accumulation. The therapeutic core of this pathway is to regulate the activity of proteins associated with VLDL assembly and secretion, thereby improving the transport efficiency of VLDL (Marigorta et al., 2024).

**FIGURE 5:**
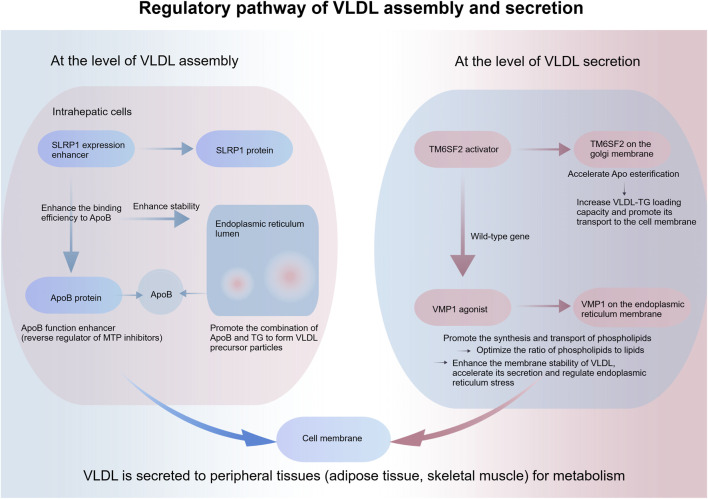
Mechanism diagram of modulators of VLDL assembly and secretion-related proteins promoting hepatic lipid transport.

Modulators of proteins related to VLDL assembly and secretion can alleviate MASLD by enhancing hepatic TG export, with the core targets and pathways summarized as follows: at the assembly level, representative agents include SLRP1 expression enhancers and ApoB function enhancers (e.g., reverse regulators of MTP inhibitors). SLRP1, as a key accessory protein for VLDL assembly, can improve its binding efficiency to ApoB when its expression is upregulated, thereby promoting the combination of ApoB with TG in the endoplasmic reticulum lumen to form VLDL precursor particles (Peyman, 2024). As the core apolipoprotein of VLDL, ApoB function enhancers can improve its stability via structural modification or degradation inhibition, enhance its TG-binding capacity, and ensure the assembly efficiency of VLDL precursors, laying a foundation for subsequent secretion. At the secretion level, TM6SF2 activators and VMP1 agonists play a pivotal role (Chen et al., 2025), TM6SF2 is localized to the Golgi membrane, and its activators can accelerate ApoB esterification, increase VLDL-TG loading capacity and promote VLDL trafficking to the cell membrane—this activation is particularly significant for the wild-type gene (which avoids the risk of the rs58542926 variant). VMP1 is localized to the endoplasmic reticulum, and its agonists can promote phospholipid synthesis and transport, optimize the phospholipid-to-lipid ratio, provide raw materials for the VLDL membrane structure to enhance particle stability and accelerate secretion, and simultaneously regulate endoplasmic reticulum stress to reduce oxidative damage to hepatocytes. The aforementioned modulators associated with assembly and secretion exert a synergistic effect, improving the efficiency of hepatic TG export and reducing lipid accumulation in hepatocytes (Jiang et al., 2022) (Figure 5).

Currently, there are no established clinical drugs that directly target the VLDL assembly and secretion pathway. However, pioglitazone and SGLT-2 inhibitors can indirectly enhance VLDL secretion efficiency and reduce hepatic triglyceride accumulation by improving hepatic lipid metabolism. Modulation of this pathway reduces hepatic TG levels and normalizes serum lipoprotein-related biomarkers. Of note, increased VLDL secretion may be accompanied by systemic dyslipidemia, necessitating monitoring based on lipid-related biomarkers such as LDL-C.

#### 4.3.1 Clinical benefits and potential risks of targeting VLDL export

Drug targeting VLDL-mediated hepatic lipid export represents a rational therapeutic strategy for MASLD, with core benefits including directly promoting hepatocellular lipid efflux and reversing hepatic steatosis (Mureddu et al., 2025). This approach provides an alternative pathway for patients with severe hepatic steatosis who do not respond to other lipid-lowering regimens. Moderate upregulation of VLDL secretion can also avoid hepatocellular lipotoxicity and reduce the risk of disease progression to NASH and hepatic fibrosis (Wang W. et al., 2025). Relevant drugs have demonstrated clear effects in reducing liver fat and improving liver function in early-phase studies, with efficacy independent of glycemic regulation, making them applicable to MASLD patients without type 2 diabetes. However, this strategy carries significant potential risks. As VLDL is a precursor to circulating LDL-C, excessive activation of its secretion may elevate serum triglyceride and LDL-C levels, exacerbating systemic dyslipidemia. Since most MASLD patients already have dyslipidemia and cardiovascular risk factors, this may further increase the likelihood of cardiovascular events such as atherosclerosis (Michalopoulou et al., 2025). It is even contraindicated in patients with familial hyperlipidemia or severe atherosclerosis. In clinical practice, a balance between benefits and risks must be achieved through moderate regulation of secretion, strict patient stratification, and combination lipid-lowering therapy. Specifically, VLDL secretion should only be restored to physiological levels, and the strategy should be limited to patients with isolated hepatic steatosis and no cardiovascular risk. For those with mild dyslipidemia, combination therapy with statins or PCSK9 inhibitors may be administered to achieve dual goals of liver protection and cardiovascular protection. Overall, this strategy is not a universal solution for MASLD but only has clinical value in specific patients with precise intervention. For those with cardiovascular risk factors, the risks outweigh the benefits. Currently, no targeted drugs have been approved, and future research should focus on developing liver-specific and moderately acting VLDL secretion modulators, as well as formulating individualized regimens based on patients’ metabolic and cardiovascular status (Wu J. Y. et al., 2025).

### 4.4 Regulating the immune and metabolic dysregulation pathway

**FIGURE 6:**
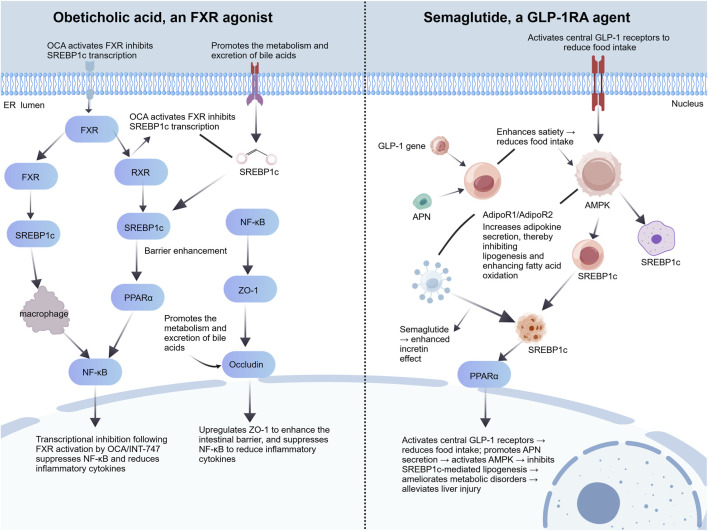
Mechanism diagram of FXR agonists and GLP-1 receptor agonists regulating immune and metabolic disorders.

Two classes of key agents can ameliorate MASLD through multiple approaches, with their specific mechanisms and clinical effects summarized as follows. First is the farnesoid X receptor (FXR) agonist obeticholic acid (OCA): as a semi-synthetic bile acid, it can highly selectively activate the nuclear receptor FXR—which is mainly expressed in hepatic and intestinal tissues and regulates bile acid metabolism, lipid metabolism and inflammatory responses (Wang et al., 2024). In terms of lipid metabolism regulation, after OCA activates FXR, FXR forms a heterodimer with RXR to repress SREBP1c transcription, block the progression DNL and reduce hepatic fatty acid and TG synthesis; it also promotes the metabolism and excretion of bile acids, which can in turn regulate PPARα expression to synergistically enhance fatty acid oxidation (Clifford et al., 2021). In inflammatory regulation, OCA activates intestinal FXR to upregulate tight junction proteins such as ZO-1 and Occludin, enhance the integrity of the intestinal barrier and reduce bacterial and endotoxin translocation; it also directly inhibits the activity of the hepatic NF-κB pathway, reduces the expression of inflammatory cytokines including TNF-α and IL-6, and alleviates hepatic inflammatory injury. Clinically, a dose of 30 mg/day can significantly reduce serum transaminase levels and liver fibrosis scores in patients, with a more pronounced efficacy in those with severe liver fibrosis. Obeticholic acid is a classic agonist of the FXR pathway. It can reduce lipid synthesis by inhibiting the SREBP1c pathway and alleviate inflammation by suppressing the NF-κB pathway. Activation of this pathway decreases serum levels of inflammatory biomarkers such as GPNMB, TNF-α, and IL-6, while upregulating bile acid metabolism biomarkers including serum tauroursodeoxycholic acid (TUDCA), thus ameliorating abnormalities in gut–liver axis-related biomarkers (Tawfiq et al., 2022). Second is semaglutide, a glucagon-like peptide-1 receptor agonist (GLP-1RA), as a long-acting GLP-1 receptor agonist, it mimics the action of endogenous GLP-1 to bind to GLP-1 receptors in relevant tissues and activate downstream pathways. In metabolic regulation, it activates central GLP-1 receptors to enhance satiety, reduce the intake of high-calorie and high-fat foods and thus lower the hepatic lipid load; it also promotes adipocyte secretion of APN, which binds to hepatocyte AdipoR1/AdipoR2 to activate AMPK—this inhibits SREBP1c-mediated lipid synthesis, promotes PPARα-mediated fatty acid oxidation, and improves insulin sensitivity to alleviate IR. In hepatoprotection, it reduces hepatic lipid accumulation and reactive oxygen species (ROS) production by ameliorating metabolic dysregulation, inhibits inflammatory cytokine expression to alleviate inflammation, and delays the progression of liver fibrosis. Clinically, a dose of 0.4 mg can lead to MASLD resolution with improved fibrosis in 59% of patients, and an average 13% weight loss is achieved simultaneously, forming a positive therapeutic cycle (Newsome et al., 2021) (Figure 6).

Liraglutide, semaglutide, tirzepatide, and other agents all target the GLP-1 pathway. Among them, tirzepatide exerts coordinated regulation via the GIP/GLP-1 dual pathway. Activation of this pathway elevates serum adiponectin levels and reduces resistin levels, downregulates hepatic GPNMB expression, attenuates abnormal inflammatory biomarkers, and normalizes serum non-coding RNA biomarkers such as miR-34a and miR-122.

## 5 MASLD-associated biomarkers

The disease spectrum of MASLD encompasses simple hepatic steatosis, metabolic dysfunction-associated steatohepatitis (MASH) and end-stage liver disease. Its clinical course is insidious and its progression exhibits heterogeneity, and conventional diagnostic methods (e.g., imaging, liver biopsy) have limitations such as insufficient early sensitivity and invasiveness (Hosseini et al., 2024). As core tools for the precise diagnosis and treatment of diseases, biomarkers enable early screening, disease stratification, efficacy evaluation and prognostic prediction of MASLD. In recent years, the discovery and mechanistic elucidation of novel biomarkers have become research hotspots. In addition to the well-identified core biomarkers, an increasing number of novel candidate biomarkers based on metabolomics, proteomics, epigenomics and the regulatory mechanism of the gut-liver axis continue to emerge, providing more abundant target support for the multidimensional diagnosis and treatment of MASLD (Lauridsen et al., 2025). The preceding text has systematically elaborated the therapeutic agents for MASLD and the core pathological pathways targeted and regulated by these drugs. As a core tool for the diagnosis and therapeutic efficacy evaluation of MASLD, the expression levels of biomarkers are directly correlated with the regulatory effects of drugs on corresponding pathways. Effective modulation of pathways including lipid metabolism and immune inflammation by pharmacologic interventions is reflected by favorable changes in the expression of specific biomarkers, whereas insufficient pathway regulation is accompanied by abnormal biomarker expression. The various biomarkers described in this section can all serve as quantitative indicators for evaluating the pathway-regulatory effects of the aforementioned drugs.

### 5.1 Specific biomarkers for the diagnosis and disease monitoring of MASLD/MASH

#### 5.1.1 GPNMB

GPNMB is a type I transmembrane glycoprotein encoded by the gene locus at 7p15, which consists of an extracellular domain, a transmembrane domain and an intracellular domain. The extracellular domain can be proteolytically cleaved and released into the systemic circulation, and GPNMB is widely expressed in adipose tissue, liver, skin and other tissues. Under the pathological conditions of MASLD/MASH, GPNMB exhibits a characteristic of specific high expression (Liu et al., 2024): transcriptomic and single-cell RNA sequencing analyses of MASH model mice revealed that its mRNA and protein levels were significantly upregulated in hepatic tissues of the disease models (*P* < 0.01), and its expression was predominantly enriched in macrophages with extremely low expression in other hepatic cells such as hepatocytes (Wang X. et al., 2025). Further preclinical animal experiments verified that GPNMB expression was markedly increased in hepatic tissues and primary hepatic macrophages of mice, and its secretory level in serum was also elevated synchronously (*P* < 0.05), regardless of the MASH model induced by a choline-deficient amino acid (CDAA) diet or the MASLD model established by a high-fat diet (HFD). These findings suggest that circulating GPNMB can serve as a non-invasive biomarker. The expression of GPNMB is directly regulated by the polarization status of macrophages: *in vitro* experiments showed that its expression was significantly downregulated when macrophages were polarized to the M1 phenotype by lipopolysaccharide (LPS), while M2 polarization induced by interleukin 4 (IL-4) could promote its expression, indicating a close association between GPNMB and the hepatic inflammatory microenvironment. Meanwhile, GPNMB acts as a metabolic crosstalk factor between the liver and adipose tissue: it can promote fatty acid synthesis in white adipose tissue, exacerbate obesity and IR, and its expression level is positively correlated with body mass index (BMI), thus building a regulatory bridge linking metabolic dysregulation and hepatic pathological changes (Fukumoto et al., 2023). Its potential clinical applications are reflected in three aspects: as a diagnostic biomarker, serum GPNMB levels can distinguish patients from healthy individuals and are positively correlated with the degree of hepatic steatosis and inflammation, making up for the deficiency of insufficient sensitivity of conventional transaminases; as a target for efficacy evaluation, its expression level can reflect changes in the polarization status of macrophages, providing a quantitative basis for the therapeutic effect of drugs; as a prognostic indicator, GPNMB-positive macrophages are associated with the progression of hepatic fibrosis, and its high expression may indicate the risk of disease progression to liver cirrhosis and even HCC, thus providing support for the stratified management of high-risk populations (Maier, 2021).

#### 5.1.2 TIMP-1

TIMP-1 is a specific inhibitor of matrix metalloproteinases (MMPs), which is mainly secreted by hepatic stellate cells, macrophages and hepatocytes. It participates in the progression of hepatic fibrosis by inhibiting the degradation of the extracellular matrix (ECM) by MMPs. In MASLD, the expression level of TIMP-1 is significantly positively correlated with the stage of hepatic fibrosis: serum TIMP-1 levels are slightly elevated in patients with simple steatosis, increase markedly in the MASH stage, and reach a peak in the cirrhosis stage. Moreover, the elevation occurs earlier than changes in liver stiffness values, making it an important indicator for the early warning of hepatic fibrosis (Wang et al., 2018).

Clinical studies have confirmed that TIMP-1 not only reflects the activation degree of hepatic stellate cells but also promotes disease progression by regulating the inflammatory microenvironment—it can inhibit the MMP activation mediated by inflammatory cytokines, leading to the abnormal deposition of ECM. Meanwhile, it enhances the expression of TNF-α and IL-6 by activating the NF-κB pathway, thus forming a vicious cycle of “inflammation-fibrosis” (Xiong et al., 2014). In addition, the combined detection of TIMP-1 and GPNMB can significantly improve the diagnostic accuracy of MASH. The two biomarkers act synergistically to reflect both hepatic inflammatory and fibrotic status simultaneously, with an area under the curve (AUC) of 0.89, which is superior to single-biomarker detection (Scarpellini et al., 2024).

#### 5.1.3 sLOX-1

LOX-1 is the primary receptor for ox-LDL, which is widely expressed in vascular endothelial cells, macrophages and hepatocytes; its soluble form, sLOX-1, can be released into the systemic circulation. In patients with MASLD, increased hepatic lipid peroxidation products lead to ox-LDL accumulation, which in turn activates the LOX-1 signaling pathway and promotes sLOX-1 release. Serum sLOX-1 levels are positively correlated with the degree of hepatic steatosis and the MASLD Activity Score (MAS) in liver tissue (Conde de la Rosa et al., 2022).

Mechanistic studies have shown that sLOX-1 can exacerbate hepatocellular injury by mediating oxidative stress responses: upon binding to ox-LDL, it activates the downstream MAPK and NF-κB pathways, induces ROS production and promotes inflammatory cytokine secretion, while simultaneously inhibiting hepatic fatty acid *β*-oxidation and exacerbating lipid accumulation (Gerhard et al., 2018). In clinical practice, post-translational modifications exhibit crucial regulatory effects on the pathological progression of MASH and are closely associated with lipid metabolism disorders in MASLD patients; the abnormal modification of key proteins related to hepatic lipid synthesis and metabolism in responders to SGLT-2 inhibitor treatment is positively correlated with the improvement of hepatic lipid deposition and the relief of liver inflammatory response (Min et al., 2025).

### 5.2 Core biomarkers for metabolic regulation and disease severity in MASLD

#### 5.2.1 Adiponectin

APN, a specific adipokine secreted by adipocytes, exists in the circulation as trimers, hexamers and high-molecular-weight complexes. It exerts biological functions by binding to AdipoR1, AdipoR2 and T-cadherin receptors in target organs, with its core regulatory pathways including the AMPK and PPARs pathways (Ghadge et al., 2018). On the one hand, it activates the AMPK pathway to promote fatty acid oxidation and inhibit DNL, thereby improving IR in the liver and peripheral tissues. On the other hand, it can activate the PPAR-α pathway via AdipoR2 to enhance the efficiency of mitochondrial fatty acid *β*-oxidation; meanwhile, it activates the PPAR-γ pathway to promote its own secretion, forming a positive regulatory cycle. In addition, it exerts anti-inflammatory effects by antagonizing TNF-α and inhibiting NF-κB activation, and can also inhibit the activation of hepatic stellate cells to delay the progression of hepatic fibrosis (Mohamed et al., 2021).

The expression level of APN is significantly negatively correlated with the severity of MASLD: serum APN levels are only slightly decreased in patients with simple hepatic steatosis, decrease markedly after progression to the MASH stage, and the degree of reduction is positively correlated with the MAS and hepatic fibrosis stage in liver tissue, reaching the lowest level in the cirrhosis stage. This is accompanied by exacerbated IR and elevated inflammatory cytokines TNF-α,IL-6. Furthermore, studies on TCM syndrome types have found that serum APN levels in MASLD patients with the spleen deficiency and phlegm-dampness syndrome are significantly lower than those in patients with other syndrome types, and are negatively correlated with HOMA-IR, reflecting the micro correlation between the pathogenesis of “root deficiency and superficial excess” and metabolic dysregulation (Wang et al., 2021).

As a core therapeutic target for the prevention and treatment of MASLD with TCM, TCM compound prescriptions and monomers can regulate its expression and downstream pathways through multiple approaches: soothing the liver prescriptions can increase serum APN levels and promote hepatic AdipoR2 expression in rats, enhancing APN signal transduction to reduce lipid deposition; resolving phlegm and removing blood stasis prescriptions can activate the APN-AMPK/PPAR-α pathway and inhibit SREBP-1c-mediated lipogenesis; TCM monomers such as ginsenoside Rb1 and hawthorn leaf flavonoids can upregulate APN expression by activating the AMPK pathway, ameliorating hepatic steatosis and IR, which provides key target support for the precision treatment of MASLD with integrated traditional Chinese and Western medicine (Li et al., 2022).

#### 5.2.2 Resistin

Resistin is an adipokine predominantly secreted by adipocytes and macrophages, whose core function is to regulate glucose and lipid metabolism as well as inflammatory responses (Kusminski et al., 2005). In patients with MASLD, serum resistin levels are significantly positively correlated with hepatic lipid content and the HOMA-IR, and the magnitude of its elevation is closely associated with disease progression; the serum resistin level in patients with simple steatosis is 20 ± 10 ng/mL, which is significantly higher than that in healthy individuals, while the level in patients with MASH reaches 29 ± 13 ng/mL, an approximate 45% increase compared with patients with simple steatosis P=0.03 . This makes resistin an important metabolic biomarker for distinguishing simple steatosis from MASH, as its expression level is significantly correlated with hepatic steatosis, portal inflammation and MASH scores (Senateş et al., 2012).

Mechanistically, resistin can block insulin signal transduction and exacerbate hepatic IR by inhibiting the phosphorylation of insulin receptor substrate-1 (IRS-1). Meanwhile, it activates the SREBP1c pathway in hepatic tissue, promotes *de novo* fatty acid synthesis, and inhibits PPARα-mediated fatty acid oxidation, leading to hepatic triglyceride accumulation (Zhou et al., 2013). In addition, resistin can induce the polarization of macrophages toward the M1 phenotype, promote the secretion of proinflammatory cytokines such as TNF-α and IL-1*β*, and aggravate hepatic inflammatory injury (Zhang C. Y. et al., 2025). Clinical studies have shown that the resistin/APN ratio has an area under the curve (AUC) of 0.85 for the diagnosis of MASH, which has greater clinical value than single biomarkers. Moreover, this ratio can predict the risk of disease progression—patients with a ratio >1.2 have a significantly increased risk of developing hepatic fibrosis within 5 years (Jiang et al., 2009).

#### 5.2.3 FGF21

FGF21 is a metabolic regulator predominantly secreted by the liver, which regulates glucose and lipid metabolism, energy expenditure and inflammatory responses by binding to fibroblast growth factor receptors (FGFRs) and the co-receptor *β*-klotho (Tillman and Rolph, 2020). In patients with MASLD, serum FGF21 levels exhibit a characteristic of compensatory elevation: in the early stage of simple steatosis, FGF21 promotes fatty acid oxidation by activating the AMPK pathway to improve IR, with a mild elevation in serum levels; as the disease progresses to MASH, hepatic sensitivity to FGF21 decreases (a state referred to as FGF21 resistance), and the body maintains metabolic homeostasis by increasing FGF21 secretion, leading to a marked elevation in serum levels, with the magnitude of elevation being positively correlated with the degree of hepatic inflammation and fibrosis (Fang et al., 2025).

### 5.3 Metabolomics-related biomarkers

Metabolomics reflects the characteristics of metabolic dysregulation in disease states by analyzing changes in small-molecule metabolites in organisms, among which biomarkers of lipidomics, amino acid omics and bile acid metabolomics have been the most extensively studied in MASLD.

#### 5.3.1 Lipidomics biomarkers

Ceramides are a class of sphingolipids containing long-chain fatty acids, whose levels are significantly elevated in hepatic tissues and serum of MASLD patients and positively correlated with the degree of hepatic fibrosis (Chaurasia and Summers, 2015). Mechanistically, ceramides impair mitochondrial function in hepatocytes, thereby suppressing fatty acid *β*-oxidation, while also promoting the activation of hepatic stellate cells and accelerating collagen deposition. Clinical investigations have verified that specific serum ceramide subtypes exhibit notable diagnostic value for MASH, and their expression levels are indicative of the risk of cardiovascular complications, with elevated levels of these subtypes associated with a significantly heightened likelihood of cardiovascular events (Yu et al., 2010).

DAGs are intermediate products of lipid metabolism that accumulate abnormally in the hepatic tissues of MASLD patients (Carli, 2024). DAGs can phosphorylate insulin receptor substrates by activating the protein kinase C (PKC) pathway to block insulin signal transduction and exacerbate IR; meanwhile, abnormal DAG accumulation can induce endoplasmic reticulum stress and promote hepatocyte apoptosis (Jornayvaz and Shulman, 2012). Serum levels of 1,3-diolein 1,3‐DAG and 1-palmitoyl-2-oleoylglycerol (PO-DAG) are positively correlated with hepatic lipid content, making them potential lipid biomarkers for the early diagnosis of MASLD.

#### 5.3.2 Amino acid omics biomarkers

Serum levels of BCAAs are significantly elevated in patients with MASLD, and the underlying mechanism is closely associated with impaired BCAA catabolism induced by IR. The liver is the primary organ for BCAA metabolism; in the setting of IR, the activity of BCAA transaminase in hepatocytes is reduced, leading to BCAA accumulation in the circulation. Meanwhile, BCAA accumulation can further exacerbate IR, forming a vicious cycle (Lee et al., 2021). Clinical studies have shown that serum BCAA levels yield an AUC of 0.81 for the diagnosis of MASLD, and the leucine/isoleucine ratio can distinguish simple steatosis from MASH, with a ratio >1.5 indicating an increased risk of MASH (Van Dijk et al., 2023).

Tryptophan metabolites (kynurenine, kynurenic acid): tryptophan is metabolized to generate metabolites such as kynurenine and kynurenic acid under the action of intestinal flora and hepatic enzymes. Intestinal dysbiosis in MASLD patients leads to impaired tryptophan metabolism, resulting in elevated serum kynurenine levels and decreased kynurenic acid levels, and the kynurenine/kynurenic acid ratio is positively correlated with the stage of hepatic fibrosis (Teunis et al., 2022). Mechanistically, kynurenine can promote the activation of hepatic stellate cells and the secretion of inflammatory cytokines by activating the aryl hydrocarbon receptor (AhR), thus accelerating the progression of hepatic fibrosis.

#### 5.3.3 Bile acid metabolomics biomarkers

TUDCA is a natural hydrophilic bile acid with anti-inflammatory, anti-apoptotic and lipid metabolism-regulating effects. Serum TUDCA levels are significantly decreased in MASLD patients, and the magnitude of the decrease is negatively correlated with hepatic lipid content and the degree of inflammation (Fukumoto et al., 2023). Mechanistically, TUDCA can reduce hepatocyte apoptosis by inhibiting endoplasmic reticulum stress; meanwhile, it activates the farnesoid X receptor (FXR) pathway, inhibits SREBP1c-mediated lipogenesis, and promotes PPARα-mediated fatty acid oxidation (Xiong et al., 2014). Clinical studies have confirmed that TUDCA supplementation can reduce serum transaminase levels and hepatic lipid content in MASLD patients, and its serum concentration can serve as a biomarker for evaluating therapeutic efficacy in disease improvement.

Lithocholic acid (LCA), the major component of secondary bile acids, is produced by the metabolic transformation of primary bile acids by intestinal flora. Intestinal dysbiosis in MASLD patients leads to increased LCA production and elevated serum LCA levels, which are positively correlated with the degree of hepatic fibrosis (Scarpellini et al., 2024). LCA promotes the secretion of inflammatory cytokines by activating the NF-κB pathway and induces ROS production in hepatocytes to exacerbate oxidative stress injury; an elevation in its levels indicates intestinal-liver axis dysfunction and the risk of disease progression (Conde de la Rosa et al., 2022).

### 5.4 Epigenetic and non-coding RNA biomarkers

Epigenetic modifications and non-coding RNAs participate in the pathogenesis of MASLD by regulating gene expression, and alterations in their levels in tissues and serum can serve as important biomarkers for disease diagnosis and condition monitoring.

#### 5.4.1 Epigenetic modification biomarkers

DNA Methylation Biomarkers: Advanced hepatic fibrosis is accompanied by widespread differential methylation of CpG sites in the genome. Notably, CPT1A, the rate-limiting enzyme mediating fatty acid oxidation, shows markedly reduced methylation levels, which induces its upregulated expression and consequently aggravates hepatic lipid accumulation. Furthermore, hypomethylation is observed in the promoter regions of genes implicated in hepatic stellate cell activation (e.g.,COL1A1,TGF‐β 1), which drives the overexpression of these genes and accelerates the progression of hepatic fibrosis (Tobaruela-Resola et al., 2024). The CPT1A methylation level in cell-free DNA (cfDNA) from serum can serve as a non-invasive diagnostic biomarker, suitable for patients with contraindications to liver biopsy.

Histone modification biomarkers, the imbalance between histone acetyltransferases (HATs) and histone deacetylases (HDACs) is involved in the pathological process of MASLD. The expression of HDAC3 is elevated in hepatic tissues of MASLD patients, which enhances histone deacetylation of lipid metabolism-related genes such as PPARα and SIRT1, inhibits their expression, and exacerbates lipid accumulation. Serum HDAC3 levels are positively correlated with hepatic lipid content, and can act as an epigenetic biomarker reflecting lipid metabolic disorder (Muangpaisarn et al., 2016).

#### 5.4.2 Non-coding RNA biomarkers

MicroRNAs are closely implicated in metabolic dysfunction-associated steatotic liver disease. miR-122 is a liver-specific miRNA that accounts for more than 70% of total miRNAs in hepatocytes and participates in the regulation of fatty acid synthesis and oxidation. Serum miR-122 levels are significantly elevated in patients with MASLD, and are positively correlated with the severity of hepatic steatosis and transaminase levels, suggesting its potential as a non-invasive biomarker for early liver injury (Tobaruela-Resola et al., 2024). MiR-34a is closely related to hepatic inflammation in nonalcoholic fatty liver disease and can be used to reflect the inflammatory status of the liver. In addition, miR-21 is abnormally expressed in MASLD and is closely associated with disease progression, thus possessing certain predictive value for the development of MASH (Muangpaisarn et al., 2016).

Long non-coding RNAs (lncRNAs), The expression of lncRNA MALAT1 is elevated in hepatic tissues and serum of MASLD patients; it sequesters miR-124 via the competing endogenous RNA (ceRNA) mechanism to relieve the inhibitory effect of miR-124 on SREBP1c, thereby promoting lipogenesis. The expression of lncRNA GAS5 is decreased, and it inhibits fatty acid oxidation by regulating the PPARα pathway; its serum levels are negatively correlated with hepatic lipid content, and can act as a reverse biomarker for disease severity. lncRNA H19 promotes the secretion of inflammatory cytokines by activating the NF-κB pathway (Xu et al., 2022).

### 5.5 Gut-liver axis-related biomarkers

Dysfunction of the gut-liver axis (including intestinal dysbiosis and intestinal barrier injury) is a crucial pathogenic mechanism of MASLD, and related metabolites as well as microbiota-derived biomarkers have become research hotspots.

#### 5.5.1 Microbiota-derived metabolites

3-succinylcholic acid is a gut microbiota-related metabolite that is negatively associated with the severity of MASLD. It can alleviate hepatic steatosis by modulating the gut microbiota structure, enhancing intestinal barrier integrity, and reducing endotoxin translocation. Gut microbial metabolites and bile acid pathways have been shown to improve hepatic lipid accumulation and inflammatory responses in MASLD (Nie et al., 2024).

#### 5.5.2 Endotoxin and intestinal barrier biomarkers

LPS and lipopolysaccharide-binding protein (LBP): Intestinal barrier injury in MASLD patients leads to the translocation of LPS released by intestinal Gram-negative bacteria into the bloodstream, resulting in elevated serum LPS levels. Meanwhile, the liver increases LBP synthesis to bind LPS. Serum LBP levels are positively correlated with the inflammatory score of hepatic tissues and with the levels of TNF-α and IL-6 in the diagnosis of MASH, and can reflect the severity of gut-derived inflammation (Song and Zhang, 2022).

Fecal calprotectin (FC), a specific biomarker of intestinal inflammation that is secreted by neutrophils. Intestinal dysbiosis in MASLD patients triggers intestinal inflammation, leading to elevated FC levels, which are positively correlated with the degree of hepatic inflammation and can indirectly reflect the inflammatory transmission status of the gut-liver axis. Clinical studies have confirmed that the combined detection of FC and serum LBP can improve the diagnostic accuracy of MASH (Bourgonje et al., 2022).

### 5.6 Combined application and clinical translation prospects of biomarkers

A single biomarker cannot balance both the sensitivity and specificity of MASLD diagnosis, and the construction of a multi-dimensional combined detection model of biomarkers is the future development direction. A detection model integrating non-coding RNAs (miR-122 + miR-34a) and metabolomics biomarkers (ceramides + BCAAs) enables the integration of early screening, disease stratification and prognosis prediction for MASLD (Yuan et al., 2024).

However, the current clinical translation of biomarkers still faces numerous challenges. Firstly, the detection methods for some biomarkers (e.g., epigenetic modifiers, lncRNAs) are complex and costly, making their clinical popularization difficult. Secondly, the absence of unified detection standards and reference thresholds leads to significant discrepancies in results across different studies. Thirdly, the validation of most biomarkers is based on small sample cohorts, with a lack of long-term follow-up data from multicenter, large-sample studies. In particular, their applicability in special populations (children and adolescents, elderly patients, and patients with comorbid cardiovascular diseases or diabetes) remains unclear (Allen et al., 2024).

Future research should focus on three aspects: Firstly, optimize detection technologies and develop low-cost, high-sensitivity detection methods to facilitate the clinical popularization of biomarkers. Secondly, establish standardized multicenter and cross-population databases to clarify the reference thresholds and clinical application scenarios of various biomarkers. Thirdly, strengthen correlational studies on the “biomarker-drug action pathway”, explore efficacy biomarkers capable of predicting drug responses (e.g., FGF21 can predict the therapeutic effect of GLP-1 receptor agonists), and realize the closed-loop management of “precision diagnosis-targeted therapy-therapeutic efficacy monitoring”. Through interdisciplinary collaboration, biomarkers will provide a more solid foundation for the individualized diagnosis and treatment of MASLD, driving the transformation of disease diagnosis and treatment from “empirical medicine” to “precision medicine” (Milani et al., 2024).

## 6 Discussion

The present study systematically summarizes the research progress on the core action pathways of therapeutic drugs for MASLD and various types of biomarkers. In light of the complex pathophysiological mechanisms of the disease, the following discussion is conducted focusing on the research and development value of drugs, the application potential of biomarkers, and the current research bottlenecks, so as to provide a reference for the precision diagnosis and treatment of the disease and subsequent related research.

The core pathological feature of MASLD is hepatic lipid metabolism disorder as the initiating factor, accompanied by multi-dimensional pathological changes such as immune-inflammatory imbalance and metabolic stress cascade reactions. Therefore, the key to pharmacological therapy lies in the precise targeting of core pathways including lipid metabolism, inflammatory regulation and fibrogenesis, so as to achieve synergistic intervention at multiple pathological links.

From the perspective of lipid metabolism regulatory pathways, SREBP1c and ChREBP, as core transcription factors for DNL, have become important targets for drug research and development (Portincasa et al., 2024). Inhibitors of the SREBP1c pathway directly suppress TG synthesis in hepatocytes by blocking its hydrolytic activation, while DGAT2 inhibitors inhibit pathway activity through a dual mechanism, providing a precise intervention strategy for MASLD with excessive lipogenesis. ChREBP pathway modulators such as vitamin D3 reduce ChREBP activity by inhibiting Kctd17 expression, which effectively blocks postprandial hyperglycemia-induced fatty acid synthesis and is particularly suitable for patients with concomitant glucose metabolism disorders. The development of these two classes of pathway inhibitors has broken the limitation of the “broad-spectrum metabolic regulation” of traditional drugs, realized precise therapy targeting DNL, and provided a new strategy for the intervention of early hepatic lipid accumulation in MASLD.

In the fatty acid oxidation and lipid transport pathways, PPARα agonists promote the catabolism of FFA by enhancing the efficiency of mitochondrial *β*-oxidation (Theys, 2024), while modulators of proteins associated with VLDL assembly and secretion ameliorate the impairment of hepatic TG export. These two classes of drugs alleviate lipid accumulation from the two dimensions of “catabolism” and “export” respectively, forming a complementary effect with lipogenesis inhibitors. Notably, non-pharmacological interventions such as intermittent fasting can also exert their effects by activating the PPARα pathway, suggesting that the combined intervention of “pharmacotherapy plus lifestyle modification” may synergistically improve therapeutic efficacy through multiple pathways, which is particularly applicable to populations intolerant to pharmacological therapy. Drugs targeting the immune and metabolic disorder regulatory pathways demonstrate the advantage of “multi-target synergy”. Obeticholic acid, an FXR agonist, not only inhibits lipogenesis but also attenuates hepatic inflammation by enhancing intestinal barrier function and inhibiting the NF-κB pathway, achieving the dual effects of “metabolic regulation and anti-inflammation” (Fuchs, 2012). GLP-1 receptor agonists, as well as GIP/GLP-1 dual-receptor and GIP/GLP-1/GCGR triple-receptor agonists, act on the fundamental driving factors of the disease by synergistically regulating glycolipid metabolism, suppressing appetite and reducing body weight. Among them, retatrutide achieves a hepatic fat content reduction of 82.4%–86%, which is far superior to traditional drugs, providing a breakthrough therapeutic option for patients with severe obesity complicated with MASLD. The development of such multi-receptor agonists is highly consistent with the pathological feature of the “metabolic disorder-inflammation-fibrosis” vicious cycle in MASLD, and exhibits broader clinical application prospects. Another class of promising targeted drugs is THR*β* agonist resmetirom. It exerts synergistic effects on lipid metabolism regulation and inflammation suppression by specifically activating THR*β* in the liver (Petta et al., 2024), and Aramchol and Aldafermin in the late-stage pipeline also provide new precise targeting directions for MASLD treatment. Phase III clinical trials have confirmed its definitive efficacy for MASH, with a favorable safety profile that avoids the off-target effects of traditional drugs. The development of this agent provides a new direction for the precise targeted therapy of MASLD, particularly suitable for MASH patients complicated with dyslipidemia (Kokkorakis et al., 2024). The targeted regulation of pathways including lipid synthesis, fatty acid oxidation, and immune inflammation by various pharmacotherapies for MASLD is accompanied by corresponding biomarker responses (Tan Y. et al., 2025). For instance, activation of the PPARα/γ pathway is reflected by elevated adiponectin and reduced ceramide levels; inhibition of the NF-κB pathway is indicated by decreased GPNMB and TNF-α levels; and activation of the AMPK pathway is associated with reduced branched-chain amino acid levels. Biomarkers not only serve as quantitative indicators for the regulatory effects of drugs on target pathways, but also facilitate the identification of drug-sensitive populations. For example, patients with abnormal FGF21 levels exhibit better responses to GLP-1 receptor agonists, providing a basis for precision medicine (Wang Y. et al., 2025).

The clinical value of MASLD biomarkers extends beyond diagnosis and prognosis to the precise linkage with drug-targeted pathways. Specific biomarkers (e.g., GPNMB) reflect the regulatory effects on immune-inflammatory pathways; metabolic biomarkers (e.g., adiponectin, branched-chain amino acids) indicate modulation of lipid metabolism and insulin resistance pathways, and epigenetic biomarkers (e.g., miR-122, CPT1A methylation) reflect long-term effects on hepatocellular injury and pathway regulation (Li et al., 2025). Future studies are warranted to further establish the precise drug–pathway–biomarker relationships, enabling the transition from pathway-targeted therapy to biomarker-guided individualized treatment (Tan Q. et al., 2025). As a core tool for the precision diagnosis and treatment of MASLD, biomarkers play an irreplaceable role in disease screening, disease stratification, therapeutic efficacy assessment and prognosis prediction. The specific biomarkers, core metabolic biomarkers and candidate biomarkers summarized in this study make up for the deficiencies of traditional diagnostic methods from multiple dimensions. GPNMB, a specific biomarker for MASLD/MASH, is highly enriched in hepatic macrophages, and its serum levels are closely correlated with the degree of hepatic inflammation and fibrogenesis, which addresses the issue of insufficient sensitivity of traditional transaminases in the early diagnosis of the disease (Lotti et al., 2025). Preclinical studies have confirmed that GPNMB expression exhibits dynamic changes with disease progression and can reflect the polarization status of macrophages, thus providing a quantitative index for the efficacy assessment of anti-inflammatory drugs and being particularly suitable for the non-invasive diagnosis and disease monitoring of MASH. As a core metabolic biomarker, APN shows a significant negative correlation between its expression levels and disease severity. It can not only distinguish simple steatosis from MASH but also serve as a key target for integrated traditional Chinese and Western medicine therapy. Chinese medicine compound prescriptions and monomers improve lipid metabolism by regulating the APN-AMPK/PPARs pathway, providing a basis for the formulation of individualized treatment regimens (Liu Y. et al., 2025). Other candidate biomarkers, such as lipid metabolism-related molecules (SREBP1c, ChREBP, PPARα), epigenetic modifiers and gut-liver axis-related metabolites, reveal the molecular characteristics of disease progression from a mechanistic perspective (Caputo et al., 2023). For instance, the methylation level of CPT1A is closely associated with lipid accumulation, and serum HDCA concentrations can reflect the metabolic homeostasis of the gut-liver axis. Further validation of such biomarkers may enable the subtyping of MASLD, providing a reference for precise targeted therapy. However, the current clinical translation of biomarkers still faces numerous challenges. First, some biomarkers have poor stability; for example, epigenetic modifiers are susceptible to environmental factors such as diet and intestinal microbiota, making them difficult to serve as independent diagnostic indicators. Second, there is a lack of unified detection standards, and discrepancies exist in the detection methods and reference thresholds of biomarkers such as GPNMB and APN across different studies, which limits their widespread application. Third, most biomarkers remain in the stage of preclinical or small-sample clinical studies, and large-sample, multicenter cohort studies are lacking to validate their sensitivity and specificity. In particular, their diagnostic value in patients with comorbid metabolic diseases remains unclear (Wu Y. et al., 2025).

Despite significant progress in research on the action pathways of therapeutic drugs and biomarkers for MASLD, notable limitations remain. In terms of drug development, the targeting specificity of existing agents needs further improvement: for example, some PPAR agonists are associated with adverse effects such as weight gain and increased fracture risk, and their efficacy in ameliorating moderate to severe hepatic fibrosis is limited. Although multi-receptor agonists like retatrutide exhibit potent weight-lowering and lipid-lowering effects, direct evidence for their ability to improve hepatic histology is lacking, and their impact on long-term prognosis requires further verification. Additionally, drug development targeting pathological processes such as intestinal dysbiosis and mitochondrial dysfunction is still in its infancy, failing to achieve comprehensive coverage of multiple disease targets (Radosavljević, 2025). In the field of biomarker research, beyond the aforementioned clinical translation challenges, strategies for the combined application of biomarkers have not yet been clearly defined. A single biomarker cannot balance diagnostic sensitivity and specificity, and in-depth exploration is still needed to establish multi-biomarker combined detection models that integrate early disease screening, disease stratification and prognosis prediction. Meanwhile, research on the correlation between biomarkers and drug action pathways is insufficient, and there is a lack of predictive efficacy biomarkers for drug response, making it difficult to achieve closed-loop management of “diagnosis-treatment-assessment” (Diamantopoulos et al., 2025). Future research should focus on the following directions: first, deepen investigations into drug mechanisms of action and develop novel targeted drugs with high specificity and low adverse effects for key pathological processes including lipid metabolism disorder, inflammatory response and fibrogenesis, with a particular focus on the development of multi-pathway synergistic inhibitors and innovative TCM preparations; second, advance the clinical translation of biomarkers, validate their efficacy through large-sample, multicenter cohort studies, establish unified detection standards and reference thresholds, and construct multi-biomarker combined diagnostic models; third, strengthen correlational research on the “pathway-drug-biomarker” axis, explore specific biomarkers capable of predicting drug efficacy, and realize an individualized diagnosis and treatment model of “precision diagnosis-targeted therapy-therapeutic efficacy monitoring”; fourth, focus on research in special populations, such as children and adolescents, elderly patients, and those with comorbid cardiovascular diseases or osteoporosis, optimize drug dosages and treatment regimens, and improve the safety and efficacy of therapy. In summary, as the most prevalent chronic liver disease worldwide, the precision diagnosis and treatment of MASLD rely on the in-depth elucidation of drug action pathways and the clinical application of high-value biomarkers. Future efforts should leverage interdisciplinary collaboration to deepen mechanistic research, break through translation bottlenecks, drive innovation in therapeutic drugs and the standardized application of biomarkers, provide more reliable diagnosis and treatment regimens for MASLD patients, and ultimately improve disease prognosis (Zhang M. et al., 2025; Priego-Parra et al., 2025).

In addition, we may integrate the hybrid modeling method of artificial intelligence (AI) and utilize the current development status of intelligent oncology empowered by computing power for the diagnosis and treatment of MASLD in the future. For example, we first screen the optimal features related to liver diseases, then build an ensemble learning model based on artificial neural networks to realize liver disease identification, and simultaneously improve the interpretability and credibility of the model through explainable AI, providing a technical scheme with both accuracy and interpretability for the intelligent diagnosis of liver diseases (Xu et al., 2026; Chowdhury et al., 2025).

## 7 Conclusion

As a highly prevalent chronic liver disease worldwide, metabolic dysfunction-associated steatotic liver disease (MASLD) is characterized by hepatic lipid metabolism disorder as the core initiating factor, accompanied by multi-dimensional pathological changes including immune-inflammatory imbalance and metabolic stress. The disease spectrum shows a progressive continuum, posing a severe threat to public health. Current research has clarified the core action pathways of various therapeutic drugs, covering the targeting of lipogenesis, improvement of fatty acid oxidation, promotion of lipid transport, and regulation of immune-metabolic disorders. Conventional drugs such as pioglitazone and metformin, as well as novel agents including SGLT-2 inhibitors and GLP-1 receptor agonists, exhibit definite efficacy in ameliorating lipid metabolism, alleviating IR and relieving hepatic inflammation through the precise regulation of relevant signaling pathways, among which multi-receptor agonists demonstrate prominent advantages of multi-target synergistic therapy. Meanwhile, specific and core metabolic biomarkers such as GPNMB and APN, together with candidate biomarkers including lipid metabolism-related molecules and epigenetic modifiers, provide important tools for early disease screening, disease monitoring and therapeutic efficacy assessment. However, several challenges remain in this field, such as insufficient targeting of drug development, significant adverse effects of some drugs, and difficulties in the clinical translation of biomarkers. Existing studies have clearly identified the core functional pathways of various therapeutic agents for MASLD, as well as the responsive patterns of corresponding specific, metabolic, and epigenetically related biomarkers following pathway modulation. This has established an integrated research paradigm whereby drugs target and regulate pathways, and biomarkers quantitatively evaluate therapeutic efficacy. Conventional agents such as pioglitazone and metformin, as well as novel multi-receptor agonists including tirzepatide and retatrutide, all induce favorable changes in biomarker expression through precise modulation of core pathways, providing an objective basis for clinical efficacy assessment. Future research needs to further deepen mechanistic investigations and develop novel targeted drugs with high specificity and low adverse effects; advance the clinical validation and standardization of biomarkers and construct multi-biomarker combined detection models; and strengthen correlational research on the “pathway-drug-biomarker” axis to realize the integration of precision diagnosis, targeted therapy and therapeutic efficacy monitoring, thereby providing more reliable diagnostic and therapeutic support for patients with MASLD.
